# A comprehensive outline of the role of non-coding RNAs in vitiligo

**DOI:** 10.1016/j.bbrep.2025.101916

**Published:** 2025-01-09

**Authors:** Fateme Sadat Feghahati, Soudeh Ghafouri-Fard

**Affiliations:** Department of Medical Genetics, Shahid Beheshti University of Medical Sciences, Tehran, Iran

**Keywords:** Vitiligo, lncRNA, circRNA, miRNA

## Abstract

Vitiligo is a common skin depigmentation condition caused by selective destruction of melanocytes. It is regarded as a polygenic disorder. In addition to protein-coding loci, non-coding regions of the genome contribute to the pathogenesis of vitiligo. A bulk of evidence highlights contribution of different classes of non-coding RNAs in this condition. Expression profile of different non-coding RNAs has been evaluated in the plasma, serum, blood cells and skin samples of patients with vitiligo. Notably, these transcripts not only partake the pathogenesis of vitiligo, but also are regarded as putative targets for prospective treatment strategies for this disorder. The current review focuses on depicting the role of miRNAs, long non-coding RNAs and circular RNAs in the etiology of vitiligo. Moreover, we discuss the shared functions of these transcripts in the pathogenesis of vitiligo and melanoma.

## Highlights

• Vitiligo is a polygenic disorder.
• Non-coding regions of the genome contribute to the pathogenesis of vitiligo.
• Expression of different non-coding RNAs has been changed in the plasma, serum, blood and skin samples of these patients.
• These transcripts are putative targets for prospective treatment strategies for this disorder.

## 1 Introduction

With a global prevalence of about 1 % [1], vitiligo is the most common skin depigmentation disorder. It is a chronic autoimmune condition characterized by selective destruction of melanocytes [2]. Generally, it is classified into four types, namely non-segmental, segmental, mixed, and unclassifiable/undetermined [3]. Since different types of vitiligo might have distinct etiologies, it is important to differentiate between these subtypes.

From a genetic viewpoint, vitiligo is regarded as a “complex disorder”, describing concurrent influences of numerous genetic risk factors and environmental causes [4]. The estimated heritability of vitiligo is 0.75–0.83 [5]. A number of genome-wide association studies have been conducted to unravel the genetic background of vitiligo [[6], [7], [8]]. Cumulatively, this type of studies has resulted in the identification of more than 50 different genetic risk loci for vitiligo [4]. So far, several protein coding genes have been found to contribute to the pathogenesis of vitiligo. Meanwhile, a bulk of evidence highlights contribution of different classes of non-coding RNAs in this condition. These transcripts have a prominent role in the precise regulation of transcriptional output of protein-coding genes that are involved in the appropriate immune function [9]. Not surprisingly, they represent the majority of cellular RNAs and are regarded as biomarkers of autoimmune disorders [9]. Their contribution to the pathogenesis of several autoimmune disorders has been reviewed by different groups [10,11]. The current review focuses on depicting the role of microRNAs (miRNAs), long non-coding RNAs (lncRNAs) and circular RNAs (circRNAs) in the etiology of vitiligo. The evidence is mostly based on expression assays in different biological samples of these patients. Moreover, a number of in silico tools have been applied for identification of the putative targets and signaling pathways that are modified by these transcripts.

## 2 miRNAs in vitiligo

miRNAs have been confirmed to be promising markers for disease detection in a wide array of disorders [12]. Their role in the pathogenesis of vitiligo has also been studied during recent years (Table 1). Expression profile of different miRNAs was evaluated in the plasma, serum, and blood cells. Moreover, skin samples of patients with vitiligo have been used as a biological sample for such purpose. For instance, miR-23a was found to be down-regulated in the plasma samples of these patients compared with controls [13]. Notably, levels of this miRNA were negatively correlated with Vitiligo Area Severity Index. Moreover, plasma levels of miR-23a could discriminate between vitiligo patients and controls with 60 % specificity and 64 % sensitivity [13].

**Table 1:** Altered expression of miRNAs in vitiligo.

microRNA	Chromosal location	Alteration in vitiligo	Type of vitiligo	Pathways	Sample type	Method	Number of cases and controls	Function	References
miR-23a	19	Downregulation	7 Segmental, 43 Non-segmental	Apoptosis-related genes CASP7, IRAK1, PRKAR1A, CASP3, TNFRSF10B, PIK3R1	Plasma	qRT-PCR	50 patients, 44 healthy individuals	Related to autoimmune diseases.	[13]
				MYD88, APAF1, PPP3R1, PIK3CB					
				Melanogenesis-related genes PRKCA, ADCY6, PLCB4, MAPK1, CTNNB1, FZD4, CALM2, GNAI3					
miR-1469	15	Upregulation	Non-segmental	CD122, IFN-γ	Plasma	qRT-PCR	30 patients, 30 healthy individuals	Plays a role as a biomarker for NSV functions as an apoptosis enhancer	[14]
miR-4437	2	Downregulation	Non-segmental	N/A	Plasma	qRT-PCR	30 patients, 30 healthy individuals	N/A	[14]
miR-21–5p	17	Downregulation	N/A	STAT3, balance between Treg and Teff cells	PBMCs	qRT-PCR	15 patients, 15 healthy individuals	Its up-regulation might protect melanocytes through regulating T effector/regulatory T cells balance via STAT3.	[15]
miR-421	X	N/A	N/A	RIPK1, PI3K/AKT/mTOR	Melanocyte	qRT-PCR	N/A	Downregulates PER K, eIF2α and CHOP protein expression	[17]
miR-155	21	Upregulation	Non-segmental	Related to melanogenesis MITF, POMC, oxidative stress defense (PRDX5), apoptosis (FAS, p53, MYC), DNA repair (NBN, p53, and ATM), autoimmunity-related genes (TNF, IL2, IL6, IL1B, IFNG, TGFBR2, HLA-C, and STAT3), and cell adhesion (ITGA5)	Plasma	qRT-PCR	100 patients, 100 healthy individuals	Inhibitsexpression of melanogenesis-associated genes (TYRP1, YWHAE, SDCBP and SOX10 in melanocytes, and YWHAE in keratinocytes) and alters interferon regulated genes (SOCS1, IRF1 and IFITM1) in melanocytes and keratinocytes stimulating Foxp3 transcription, Related to MIFT	[16]
miR-9	1	Upregulation	Non-segmental		Plasma	qRT-PCR	100 patients, 100 healthy individuals	Related to expression SIRT1, Ecadherin and β1 integrin	[16]
miR-124	20	Upregulation	Non-segmental		Plasma	qRT-PCR	100 patients, 100 healthy individuals	Related to RACK1	[16]
miR-181a	1	Upregulation	Non-segmental		Plasma	qRT-PCR	100 patients, 100 healthy individuals	Roles in multiples physiological processes in the skin, including keratinocytes proliferation, melanogenesis, wound healing, and skin ageing	[16]
miR-7	9	Upregulation	Non-segmental		Plasma	qRT-PCR	100 patients, 100 healthy individuals		[16]
miR-23 b	9	Upregulation	Non-segmental		Plasma	qRT-PCR	100 patients, 100 healthy individuals		[16]
miR-145	5	Downregulation	Non-segmental		Plasma	qRT-PCR	100 patients, 100 healthy individuals		[16]
miR-148a	7	Upregulation	Non-segmental		Plasma	qRT-PCR	100 patients, 100 healthy individuals		[16]
miR-148 b	12	Upregulation	Non-segmental		Plasma	qRT-PCR	100 patients, 100 healthy individuals		[16]
miR-203a	14	Upregulation	Non-segmental		Plasma	qRT-PCR	100 patients, 100 healthy individuals		[16]
miR-320a	8	Upregulation	Non-segmental		Plasma	qRT-PCR	100 patients, 100 healthy individuals		[16]
miR-423–5p	17	Upregulation	Non-segmental	Involved in proteoglycans in cancer, Hippo signaling pathway, fatty acids metabolism, proteins processing in the endoplasmic reticulum, adherens junction, endocytosis, fatty acids synthesis, and biosynthesis of unsaturated fatty acids	Plasma	qRT-PCR	85 patients, 85 healthy individuals	Involved in the melanogenesis pathway	[18]
				Related to CREB1					
miR-182–5p	7	Upregulation	Non-segmental	Related to CREB1, PTEN	Plasma	qRT-PCR	85 patients, 85 healthy individuals	–	[18]
miR-106a	X	Upregulation	Non-segmental	Related to CREB1, PTEN	Plasma	qRT-PCR	85 patients, 85 healthy individuals	Acts as a tumor suppressor in melanoma by E2F3	[18]
								Related to autoimmune skin disorder, psoriasis	
miR-130a	11	Upregulation	Non-segmental	Related to Wnt/β-catenin signaling	Plasma	qRT-PCR	85 patients, 85 healthy individuals	Role in cancer and autoimmune diseases	[18]
miR-152	17	Upregulation	Non-segmental	Involved in proteoglycans in cancer, Hippo signaling, fatty acid metabolism, proteins processing in the endoplasmic reticulum, adherens junction, endocytosis, fatty acids biosynthesis, and synthesis of unsaturated fatty acids	Plasma	qRT-PCR	85 patients, 85 healthy individuals	Role in tumorigenesis and autoimmune diseases	[18]
miR-137	1	No different	Non-segmental		Plasma	qRT-PCR	85 patients, 85 healthy individuals	Inhibits melanogenesis in mouse skin melanocytes through suppression of c-KIT and TYRP2 in the SCF/c-KIT pathway	[18]
miR-374 b-5p	X	No different	Non-segmental		Plasma	qRT-PCR	85 patients, 85 healthy individuals	Involved in the melanogenesis pathway	[18]
miR-196 b-5p	7	No different	Non-segmental		Plasma	qRT-PCR	85 patients, 85 healthy individuals	Involved in the melanogenesis pathway	[18]
miR-211	15	–	–	–	Blood	PCR–RFLP	136 patients, 129 healthy individuals	miR-211 rs8039189 polymorphism reduced risk of vitiligo.	[19]
miR-211	15	Downregulation	–	–	Skin	RNA seq	5 patients (non-lesional and lesional epidermis)	Down-regulation of miR-211–5p level in the lesional epidermis of patients with vitiligo was most likely mediated by MALAT1 in a reciprocal process.	[20]
miR-211	15	Downregulation	–	Has a role in the regulation of oxidative stress and energy metabolism in mitochondria, participates in the melanin homeostasis	Skin	qRT-PCR	11 patients, 5 healthy individuals	N/A	[21]
miR-1238	19	–	58 Segmental,	N/A	Blood	PCR–RFLP	136 patients, 129 healthy individuals	No significant association was detected between the rs12973308 of miR-1238 and risk of vitiligo.	[19]
			5 Non-segmental,						
			73 Focal						
miR-1238	19	Downregulation	N/A	N/A	Peripheral blood	miRNA array, qRT-PCR	5 patients, 5 healthy individuals	Axon guidance pathway was most considerably associated with this miRNA.	[22]
miR-202	10	–	58 Segmental,	Related to anti-inflammatory cytokine IL-10	Blood	PCR–RFLP	136 patients, 129 healthy individuals	miR-202 rs12355840 polymorphism was correlated with increased risk of vitiligo.	[19]
			5 Non-segmental,						
			73 Focal						
miR-202	10	Up-regulation			Peripheral blood	miRNA array, qRT-PCR	5 patients, 5 healthy individuals	–	[22]
miR-493	14	Upregulation	Segmental	Related to HNRNPU/COMT/dopamine axis	Plasma	qRT-PCR	7 patients, 8 healthy individuals	The increase in dopamine due to miR-493–3p upregulation leads to heightened ROS production and melanocyte apoptosis.	[23]
miR-224–3p	X	Upregulation	Non-segmental	N/A	PBMC Skin	Microarray	60 patients, 60 healthy individuals	Role in regulation of inflammatory cytokine production	[24]
						RT-PCR			
						qRT-PCR			
miR-183–5p	7	Upregulation	_	N/A	Skin	qRT-PCR	Mice	Directly regulates MITF in iMC23 melanocytes	[25]
miR-200c	12	Downregulation	N/A	Enhances the levels of melanogenesis-related genes through inhibiting SOX1 to activate β-catenin	Skin	qRT-PCR	6 patients, 10 healthy individuals	N/A	[26]
miR-125 b-5p	11	Upregulation	N/A	Affecting cell proliferation, cell cycle progression, apoptosis, and melanogenesis	Serum	qRT-PCR	17 patients, 17 healthy individuals	Regulates biological behaviors of melanocytes and melanogenesis by downregulating MITF level	[27]
miR-146a	5	Upregulation	Non-segmental	miR-146a expression was upregulated, whereas Foxp3 expression was downregulated.	PBMC	Microarray	50patients, 25healthy individuals	Considered as an essential regulator of posttranscriptional gene expression, has a potential role in autoimmune diseases,	[28]
						RT-PCR		Regulates Foxp3 transcription factor, thus affecting function of regulatory T cells	
miR-25	7	Upregulation	Non-segmental	Inhibits SCF and bFGF production and secretion from keratinocytes, compromising their protective paracrine effect on melanocytes under oxidative stress	Serum	qRT-PCR	80 patients, 80 healthy individuals	Xacerbates H2O2-induced melanocyte destruction and dysfunction	[29]
miR-377	14	Upregulation	N/A	Dysregulated miR-377 may contribute to vitiligo pathogenesis through downregulation of PPAR-γ and upregulation of IL-17.	Serum	qRT-PCR	30 patients,	N/A	[30]
							30 healthy individuals		
miR-766–3p	X	Downregulation	N/A	N/A	Peripheral blood	miRNA array, qRT-PCR	5 patients,	N/A	[22]
							5 healthy individuals		
miR-630	15	Downregulation	N/A	N/A	Peripheral blood	miRNA array, qRT-PCR	5 patients,	N/A	[22]
							5 healthy individuals		
Let7a	9	Up-regulation	–	–	Skin biopsy exosome	qRT-PCR	50 patients with vitiligo vulgaris, 50 healthy individuals	Expression of let7a was correlated with disease duration.	[31]

Expression profile of miRNAs was also assessed in plasma exosomes of patients with vitiligo. For instance, using the high-throughput sequencing, Wei et al. found that miR-1469 was significantly up-regulated in circulating exosomes of patients with non-segmental vitiligo [14]. Authors then assessed the effects of these circulating exosomes on NK cells and reported elevation of NK cell proliferation ability and IFN-γ secretion ability following this treatment [14]. Moreover, additional experiments demonstrated that upregulation of the miR-1469 target CD122 could partly converse the effect of miR-1469 on these cells [14]. Cumulatively, authors suggested that abnormal levels of in plasma exosomal cargo may participate in the dysfunction of NK cells in this type of vitiligo [14]. Additionally, exosomal miR-1469 was suggested as a biomarker of disease activity and a target for manipulation of innate immune responses in the non-segmental vitiligo [14].

**Fig. 1:**
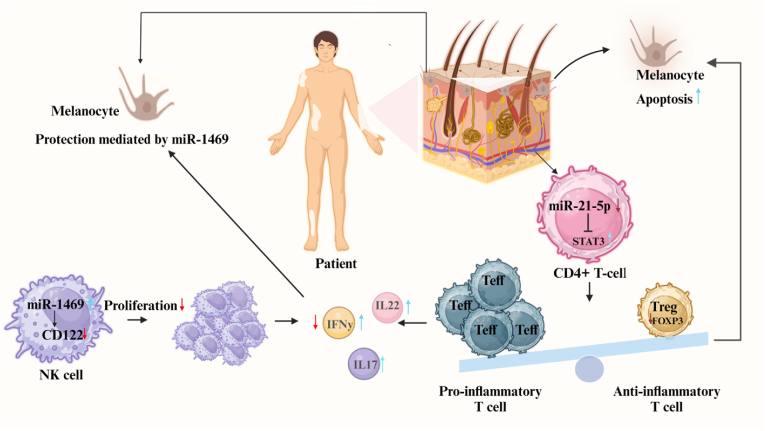
Mechanisms of the interaction between miR-21–5p and STAT3 and interaction between miR-1469 and CD122 in vitiligo. Down-regulation of miR-21–5p induces the differentiation of CD4^+^ T cells towards Teff cells, leading to pro-inflammatory responses and thereby inducing apoptosis in melanocytes. miR-1469 acts as a protective factor that is remarkably upregulated as a result of overactivation of NK cells at initial and progressive phases, thus hindering the activity of NK cells and overturning the activity of vitiligo, consequently preserving the balance of the immune system. miR-1469 could decrease NK cell proliferation and IFN-γ production through suppression of CD122.

Expression assays in peripheral blood mononuclear cells (PBMCs) revealed down-regulation of miR-21–5p and up-regulation of STAT3 expressions in patients with vitiligo compared with controls [15]. Functional analyses demonstrated that miR-21–5p upregulation in Th17-polarized CD4^+^ T cells resulted in the reduction of percentage of effector T cells and associated cytokines, such as IL-17 A and IL-22, while enhanced the percentage of regulatory T cells and Foxp3 [15]. Notably, co-culture of melanocytes with Th17-polarized CD4^+^ T cells in the presence of a miR-21–5p mimic led to apoptosis of melanocytes [15]. Taken together, miR-21–5p may have a protective effect on melanocytes through targeting STAT3 and regulating the balance between regulatory and effector T cells [15]. Fig. 1 depicts mechanisms of actions of miR-1469 and miR-21–5p in the development of vitiligo.

Finally, expression assay of 11 immune-related circulating miRNAs in plasma samples of patients with psoriasis and vitiligo revealed up-regulation of all miRNAs in both conditions as compared with normal controls [16]. Notably, these miRNAs were more prominently up-regulated in psoriasis compared with vitiligo. Expressions of the majority of miRNAs were correlated with each other, proposing common targets and/or pathways [16]. Besides, all assessed miRNAs exhibited capacity as biomarkers for autoimmune skin diseases, with miR-145 being the most appropriate one [16]. Other miRNAs involved in vitiligo and their potential pathways are shown in Table 1.

## 3 lncRNAs in vitiligo

A number of studies simultaneously evaluated expression of a candidate lncRNA and a miRNA that is potentially sponged by this lncRNA in biological samples of patients with vitiligo [30,32]. For instance, Alhelf et al. measured serum levels of lncRNA TUG1, miR-377 and other related genes in these patients and corresponding controls [30]. They reported down-regulation of lncRNA TUG1 and PPAR-γ levels, parallel with up-regulation of miR-377 and IL-17 in the vitiligo group compared with the controls [30]. They suggested that mentioned non-coding RNAs possibly partake in the pathogenesis of vitiligo through downregulation of PPAR-γ and upregulation of IL-17 [30].

Another type of study performed by other groups was based on high-throughput RNA sequencing. This approach led to identification of 292 differentially expressed lncRNAs between non-segmental vitiligo subjects and controls [33]. Differential expression of six lncRNAs was also assessed by qRT-PCR, demonstrating upregulation of ENST00000460164.1 and NR-046211.1 in PBMCs of non-segmental vitiligo [33]. Moreover, authors constructed an lncRNA-miRNA-mRNA network comprising two lncRNAs, 17 miRNAs, and 223 mRNAs [33]. Similarly, microarray analysis was used to compare expression levels of different transcripts in T cells of patients with vitiligo versus controls [34]. Among the differentially expressed lncRNAs among T cells was LOC100506314 whose expression was enhanced in vitiligo, particularly in CD4^+^ T cells, but not in CD8^+^ T cells [34]. The expression level of this lncRNA in CD4^+^ T cells was meaningfully associated with the severity of vitiligo [34]. LOC100506314 was found to bind with STAT3 and MIF [34]. Overexpression of LOC100506314 suppressed the phosphorylation of STAT3, AKT, and ERK. In addition, it decreased levels of nuclear protein of p65, IL-6 and IL-17 in Jurkat cells and T cells of patients with vitiligo [34]. Table 2 shows altered expression of lncRNAs in vitiligo.

**Table 2:** Altered expression of lncRNAs in vitiligo.

lncRNA	Chromosal location	Alteration in vitiligo	Type of vitiligo	Pathways	Sample type	Method	Number of cases and controls	Function	References
H19	11	Up-regulation	–	–	Skin biopsy exosome	qRT-PCR	50 patients with vitiligo vulgaris, 50 healthy individuals	Expression of H19 was correlated with the disease activity.	[31]
MALAT1	11	Upregulation	Non-segmental	MALAT1–miR-211–SIRT1	Skin	RNA-seq	5 patients (non-lesional and lesional epidermis)	MALAT1 enhanced expression of SIRT1 and a concomitant removal of UVB-induced CPDs in primary keratinocytes.	[20]
ENST00000460164.1	14	Upregulation	Non-segmental	Associated with metabolism, apoptosis, Jak-STAT, and PI3K-Akt signaling pathways	PBMCs	qRT-PCR	30 patients, 30 healthy individuals	N/A	[33]
NR-046211.1	14	Upregulation	Non-segmental	Associated with metabolism, apoptosis, Jak-STAT, and PI3K-Akt signaling pathways	PBMCs	qRT-PCR	30 patients, 30 healthy individuals	N/A	[33]
ENST00000393264.2	22	No different	Non-segmental	_	PBMCs	qRT-PCR	30 patients,	N/A	[33]
							30 healthy individuals		
NR-135491.1	_	No different	Non-segmental	_	PBMCs	qRT-PCR	30 patients, 30 healthy individuals	N/A	[33]
NR-135320.1	13	No different	Non-segmental	_	PBMCs	qRT-PCR	30 patients,	N/A	[33]
							30 healthy individuals		
ENST00000381108.3	X	No different	Non-segmental	_	PBMCs	qRT-PCR	30 patients, 30 healthy individuals	N/A	[33]
Lnc-ARRDC3-1	17	Upregulation	non-segmental	N/A	T-cell	Microarray Analysis, RT-PCR	41 patients, 41 healthy individuals	N/A	[34]
PLCG1	20	Upregulation	Non-segmental	N/A	T-cell	Microarray Analysis, RT-PCR	41 patients,	N/A	[34]
							41 healthy individuals		
A_33_P3229958	1	Upregulation	Non-segmental	N/A	T-cell	Microarray Analysis, RT-PCR	41 patients, 41 healthy individuals	N/A	[34]
TM4SF19	3	downregulation	Non-segmental	N/A	T-cell	Microarray Analysis, RT-PCR	41 patients,	N/A	[34]
							41 healthy individuals		
IFI27	14	Downregulation	Non-segmental	N/A	T-cell	Microarray Analysis, RT-PCR	41 patients,	N/A	[34]
							41 healthy individuals		
IL17RB	3	Downregulation	Non-segmental	N/A	T-cell	Microarray Analysis, RT-PCR	41 patients,	N/A	[34]
							41 healthy individuals		
TREM1	6	Upregulation	Non-segmental	N/A	T-cell	Microarray Analysis, RT-PCR	41 patients,	N/A	[34]
							41 healthy individuals		
RAB13	1	Upregulation	Non-segmental	N/A	T-cell	Microarray Analysis, RT-PCR	41 patients,	N/A	[34]
							41 healthy individuals		
LOC100506314	12	Upregulation	Non-segmental	Interacted with STAT3 and MIF and inhibited IL-6 and IL-17 expression by suppressing the STAT3, NF-κB, AKT, and ERK pathways	T-cell	Microarray Analysis, RT-PCR	41 patients,	Increased in vitiligo, especially CD4^+^, but not CD8^+^ T cells	[34]
							41 healthy individuals		
TUG1	22	Downregulation	Non-segmental-28	TUG1 associated with PPAR-c downregulation and IL-17 upregulation and MAPK pathway	Serum	qRT-PCR	30 patients,	N/A	[30]
			Segmental-2				30 healthy individuals		

## 4 circRNAs in vitiligo

Most of studies that assessed expression of circRNAs in vitiligo have used high throughput techniques (Table 3). For instance, using microarray technique, Li et al. have identified 64 dysregulated circRNAs in skin samples of these patients in addition to 14 dysregulated miRNAs [35]. Correlation analyses have led to construction of 48 correlated axes in the circRNA/miRNA/mRNA regulatory network. Further analyses have shown enrichment of these circRNAs in ubiquitin mediated proteolysis, endocytosis and RNA degradation, and Jak/STAT pathway [35].

**Table 3:** Altered expression of circRNAs in vitiligo.

circRNA	Chromosal location	Alteration in vitiligo	Type of vitiligo	Pathways	Sample type	Method	Number of cases and controls	Function	References
hsa_circ_0007716	17	Upregulation	Non-segmental	Regulate SEMA4D, ATG13, and ANKRD6	Skin tissue	qRT-PCR	6 patients, 6 healthy individuals	Regulation of physiology and pathology of vitiligo	[35]
hsa_circ_0003164	14	Upregulation	N/A	N/A	Skin tissue	qRT-PCR	6 patients, 6 healthy individuals	N/A	[35]
hsa_circ_0028899	12	Downregulation	N/A	N/A	Skin tissue	qRT-PCR	6 patients, 6 healthy individuals	N/A	[35]
hsa_circ_0087961	9	–	Non-segmental	N/A	Peripheral blood	Microarray	4 patients (before and after treatment with methylprednisolone)	Regulation of physiology and pathology of vitiligo	[36]
ciRS-7	N/A	N/A	Non-segmental	Related to miR-7/STAT3 and AKT/FGF2	Peripheral blood	Microarray	4 patients (before and after treatment with methylprednisolone)	Expression in HaCaT, PIG1, MNT1, MC, and FB cells	[36]
circ_0091223	X	N/A	Non-segmental	Related to cAMP/PKA	Peripheral blood	Microarray	4 patients (before and after treatment with methylprednisolone)	Important role in melanogenesis. Potential biomarker for skin pigmentation disorders	[36]
hsa_circ_0048910	19	N/A	Non-segmental	Related to oxidative stress genes	Peripheral blood	Microarray	4 patients (before and after treatment with methylprednisolone)	Oxidative stress injury of melanocytes	[36]
				SOD2, PTGS2, DHFR, HMOX1, FOSL1, and PARP1					
hsa_circ_0048909	19	N/A	Non-segmental	Related to oxidative stress genes SOD2, PTGS2, DHFR, HMOX1, FOSL1, and PARP1	Peripheral blood	Microarray	4 patients (before and after treatment with methylprednisolone)	Oxidative stress injury of melanocytes	[36]
hsa_circRNA_100842	N/A	Downregulation	Non-segmental	Related to ferroptosis pathway (SLC3A2)	N/A	Microarray	4 patients (before and after treatment with methylprednisolone)	N/A	[36]
hsa_circRNA_100283	N/A	Downregulation	Non-segmental	Related to ferroptosis pathway (GCLM)	N/A	Microarray	4 patients (before and after treatment with methylprednisolone)	N/A	[36]
hsa_circRNA_101076	N/A	Downregulation	Non-segmental	Related to ferroptosis pathway (PCBP2)	N/A	Microarray	4 patients (before and after treatment with methylprednisolone)	N/A	[36]
hsa_circRNA_103556	N/A	Downregulation	Non-segmental	Related to ferroptosis pathway (TFRC)	N/A	Microarray	4 patients (before and after treatment with methylprednisolone)	N/A	[36]
hsa_circRNA_104600	N/A	Downregulation	Non-segmental	Related to ferroptosis pathway (VDAC3)	N/A	Microarray	4 patients (before and after treatment with methylprednisolone)	N/A	[36]
circBub1b	N/A	Upregulation	Non-segmental	N/A	Skin tissue	qRT-PCR	N/A	–	[37]
circTmem26	N/A	Upregulation	Non-segmental	N/A	Skin tissue	qRT-PCR	N/A	–	[37]
hsa_circRNA_000957	19	Downregulation	Segmental, Non-segmental	Related to growth hormone receptor signaling pathway, thyroid hormone response, steroid hormone response, signaling pathway, sphingolipid metabolism, and essential amino acid biosynthesis	Skin tissue	qRT-PCR	5 patients, 5 healthy individuals	N/A	[38]
hsa_circRNA_101798	16	Upregulation	Segmental, Non-segmental	Related to growth hormone receptor signaling pathway, thyroid hormone response, steroid hormone response, signaling pathway, sphingolipid metabolism, and essential amino acid biosynthesis	Skin tissue	qRT-PCR	5 patients, 5 healthy individuals	N/A	[38]

Zhang et al. have assessed expression of circRNAs in vitiligo patients prior to and after treatment with methylprednisolone [36]. They have reported differential expression of 375 circRNAs (51 upregulated and 324 downregulated) [36]. Notably, these circRNAs have been found to be enriched in vitiligo-associated processes, including ferroptosis, organic substances transport, protein metabolic processes, and cellular components organization or biogenesis [36].

## 5 Shared miRNAs between vitiligo and skin cancer

Associations between vitiligo and melanoma have been recognized for a long time. Melanoma-associated leukoderma happens in a proportion of melanoma patients correlated with better prognoses [39]. In addition, the incidence of melanoma-associated vitiligo after treatment of melanoma is considered to associate with favorable outcome [40]. Notably, we found that several dysregulated miRNAs in vitiligo are involved in the pathogenesis of skin cancer, particularly melanoma (Table 4).

**Table 4:** miRNAs that are involved in the pathogenesis of both vitiligo and melanoma.

microRNA	Role in skin cancer	Reference
miR-23a	miR-23a-3p binds to 3′UTR of KLF3 and reduces KLF3 expression. Up-regulation of KLF3 decreases the effect of miR-23a-3p on melanoma development.	[32]
miR-1469	miR-1469 up-regulation inhibits the migration and invasive ability of melanoma cell lines. Thus, its downregulation contributes to disease progression and metastases in melanoma.	[41]
miR-21–5p	miR-21–5p promotes the growth of melanoma cells by targeting CDKN2C, which can induce cell cycle arrest at G0/G1 phase.	[42]
miR-155	miR-155 is down-regulated in melanoma samples compared to non-cancer samples. Up-regulation of miR-155 was associated with superficial spreading of melanoma and high mitotic rate.	[43]
miR-9	Up-regulation of miR-9 suppresses proliferation, self-renewal, migration, and tumorigenicity of melanoma cells and alters expression levels of BRAF, some EMT factors, and stemness genes.	[44]
miR-124	miR-124 is down-regulated in melanoma tissues compared with normal skin tissues, parallel with up-regulation of RACK1. miR-124 inhibits expression of RACK1; inhibits proliferation, migration, and invasion of melanoma cells, and promotes melanoma cell apoptosis.	[45]
miR-145	miR-145 in melanoma, contrary to many other tumors, does not essentially function via the target, FSCN1, thus the downregulation of FSCN1 does not inhibit cell proliferation or migration but, in contrast, increases cell invasion.	[46]
miR-148	Expression of miR-148a is regulated by DNA methylation and targeted by TGIF2. Its methylation is regarded as a potential prognostic marker in skin cancer.	[47]
miR-320a	Targeted binding of miR-320a to PBX3 protein inhibits the malignant phenotypes of cells and affects the occurrence and development of melanoma.	[48]
miR-137	miR-137 acts as a tumor suppressor in malignant melanoma. It regulates expression of c-Met, YB1, EZH2, and MITF.	[49]
miR-493	miR-493 is a tumor suppressor and suppresses cell proliferation and cell cycle in human melanoma through targeting IRS4.	[50]
miR-224	miR-224–5p inhibits melanoma growth and metastasis. miR-224–5p/PAK4-mediated CRAF/MEK/ERK is a potential treatment target in melanoma.	[51]
miR-183	MALAT1/miR-183/ITGB1 axis is involved in the pathogenesis of melanoma.	[52]
miR-200c	miR-200c down-regulates BMI-1, ABCG2, ABCG5, and MDR1 expressions and increases E-cadherin levels.	[53]
miR-125 b	miR-125 b directly represses stress-responsive MAPK genes and indirectly extends activated EGFR signaling through suppressing Vps4B.	[54]
miR-25	miR-25 promotes M14 cell proliferation and migration and enhances activity of the PI3K/Akt/mTOR signaling.	[55]
miR-377	miR-377negatively regulates E2F and MAP3K7/NF-kB pathway in melanoma cells.	[56]

## 6 Discussion

Vitiligo is an autoimmune disease that results from an intricate link between programming and functions of the immune system, features of the melanocyte autoimmune targets, and dysregulation of the immune responses [4]. In addition, abnormally expressed miRNAs are enriched in oxidative stress response, a biological process that is prominently involved in the pathogenesis of vitiligo [57]. While miR-9 and miR-211 contribute to oxidative stress response [21], miR-21–5p [15], and miR-224–3p [24] are examples of miRNAs that are involved in immune homeostasis. Finally, miR-211 [21] and miR-23a [13] possibly regulate melanin production.

Recent evidence has also shown neuronal factors as the other aspect of vitiligo pathogenesis which act in cooperation with the immune and genetic factors [58]. While several theories have been suggested for its pathogenesis, no individual factor can exclusively explain the complex etiology of vitiligo [59]. However, non-coding RNAs have been proposed as potential elements that participate in this complex network, since they can regulate almost all mentioned processes in the vitiligo pathogenesis [59]. Among different aspects of vitiligo pathogenesis, oxidative stress, autoimmunity, and melanocyte biology have been proved to be modified by non-coding RNAs [59]. Besides, exosomal miRNAs derived from immune cells or keratinocytes have been confirmed to participate in different aspects of vitiligo pathogenesis [14,23,26]. Based on the momentous clinical implication of miRNAs in vitiligo, specific targeting of exosomal miRNAs has been suggested as a possible approach for treatment of this disorder [60]. This is due to feasibility of *in vitro* manipulation of exosomes. Application of anti-miRNAs for the purpose of counteracting the over-activation of miRNAs is a possible strategy for treatment of vitiligo. Meanwhile, miRNA replacement using miRNA mimics or virus-mediated miRNA gain-of-function has been suggested as a tool for reintroducing a certain miRNA that is down-regulated in the affected tissues. An *in vitro* example of such approach has been provided by using miR-211 mimics that could enhance the migratory ability of melanocytes via the p53-TRPM1/miR-211-MMP9 axis and increase melanin levels [61].

It is worth mentioning that several non-coding RNAs act in concert to affect a certain process that is involved in the vitiligo. This is mostly true for lncRNAs and circRNAs that act as molecular sponges for miRNAs, thus reducing their bioavailability and regulating expression of miRNA targets.

Identification of miRNAs that regulate both vitiligo pathogenesis and melanoma carcinogenesis is an important finding of the current literature search study, since that can be used as biomarkers for prediction of melanoma course or occurrence of therapy-associated vitiligo. These miRNAs are commonly involved in the regulation of melanocyte functions and immune responses. In fact, the basic processes involved in melanoma-associated vitiligo and vitiligo might be common. Identification of these processes would facilitate finding new markers and therapeutic targets for both conditions [40].

Most of reviewed studies have focused on non-segmental vitiligo, leaving the segmental vitiligo less studied. Since the etiology of these two types of vitiligo might be different, it is necessary to conduct further comparative studies in both types of vitiligo to find distinctive biological functions of non-coding RNAs in these subtypes. Moreover, future studies should compare the expression of earlier biomarkers of non-segmental and segmental vitiligo with different classes of non-coding RNAs to explore the mechanistical issues of dysregulation of non-coding RNAs in vitiligo.

## CRediT authorship contribution statement

**Fateme Sadat Feghahati:** Formal analysis, Data curation. **Soudeh Ghafouri-Fard:** Writing – review & editing, Writing – original draft, Supervision, Conceptualization.

## Ethics approval and consent to participate

Not applicable.

## Consent for publication

Not applicable.

## Availability of data and material

Not applicable.

## Funding

No funding was received.

## Declaration of competing interest

Authors declare no conflict of interests.
